# *Rickettsia felis* in *Ctenocephalides* spp*.* Fleas, Brazil

**DOI:** 10.3201/eid0803.010301

**Published:** 2002-03

**Authors:** Riva P. Oliveira, Márcio A.M. Galvão, Claudio L. Mafra, Chequer B. Chamone, Simone B. Calic, Sergio U. Silva, David H. Walker

**Affiliations:** * Universidade Federal de Ouro Preto, Minas Gerais State, Brazil; † Fundação Ezequiel Dias, Minas Gerais State, Brazil; ‡ Secretaria Municipal de Saúde de Coronel Fabriciano, Minas Gerais State, Brazil; § University of Texas Medical Branch, Galveston, Texas, USA

**Keywords:** Rickettsia felis, Ctenocephalides spp., flea, spotted fever group rickettsiosis

## Abstract

In June 2000, suspected cases of Brazilian spotted fever (BSF) occurred in Coronel Fabriciano Municipality, Minas Gerais State, Brazil. Pooled fleas collected near two fatal cases contained rickettsial DNA. The nucleotide sequence alignment of the 391-bp segment of the 17-kDa protein gene showed that the products were identical to each other and to the *R. felis* 17-kDa gene, confirming circulation of *R. felis* in Brazil.

The pathogenic rickettsiae are a group of intracellular bacteria responsible for various human diseases. *Rickettsia rickettsii* and *R. typhi* and the diseases they cause—Brazilian spotted fever (BSF), transmitted by the *Amblyomma cajennense* tick, and murine typhus, transmitted by the Oriental rat flea—have been recognized in Brazil since the 1920s (*1*–*3*). Molecular methods, including detection by DNA amplification by polymerase chain reaction (PCR) and DNA sequence analysis, are useful in characterizing rickettsial agents in arthropods. This approach has allowed the identification of new species, such as *R. felis* in opossums, fleas (*4*,*5*), and blood and skin from ill humans from the United States, Mexico, France, and Brazil (*6*–*9*). We report the identification of *R. felis* in *Ctenocephalides* fleas collected during the investigation of an outbreak of spotted fever group rickettsiosis in Brazil.

## Material and Methods

**Figure 1:**
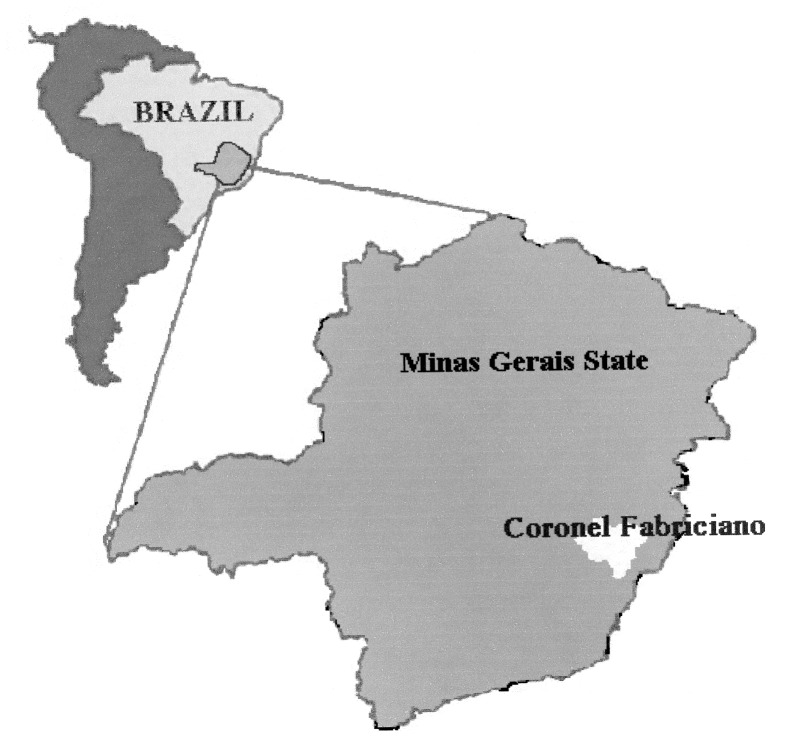
Map of Brazil and Minas Gerais State, showing Coronel Fabriciano municipality.

In June 2000, fleas and ticks were collected in a periurban area of the city of Coronel Fabriciano, Steel Valley, Minas Gerais State, Brazil (Figure 1). This survey was performed during an outbreak of suspected BSF in which two children died. They were brothers who lived in the same house. The first child who became ill was 12 years old; during the course of his disease he had fever, nausea, vomiting, diarrhea, abdominal pain, headache, myalgia, and edema. Later, renal failure and stupor occurred. The second patient had fever, rash, nausea, vomiting, diarrhea, abdominal pain, headache, myalgia, jaundice, and renal failure. Both patients reported a tick bite a day before the osnet of disease. One death was later confirmed as a case of spotted fever group rickettsiosis by immunohistochemical technique in tissues collected at autopsy. PCR was performed on brain, stomach, liver, spleen, and kidney tissues collected at autopsy, preserved in formalin, and sent to the University of Texas Medical Branch at Galveston. Because the DNA was not preserved, the death could not be attributed specifically to *R. rickettsii*, *R. felis,* or other species of *Rickettsia*.

The ticks were collected from three dogs and five horses near the house where the deaths occurred and were stored in 70% ethanol at room temperature. Ticks were separated into 15 pools with three specimens per pool, undifferentiated by life stage or sex. Fleas were also removed from 10 dogs in the home of the child whose death was confirmed as being due to BSF (Galvão et al., unpub. data) and from 3 cats near this residence; fleas were stored at –70°C. The fleas were separated into six pools with five specimens per pool. The ticks and fleas were identified as *A. cajennense* and *Ctenocephalides* spp., respectively.

**Figure 2:**
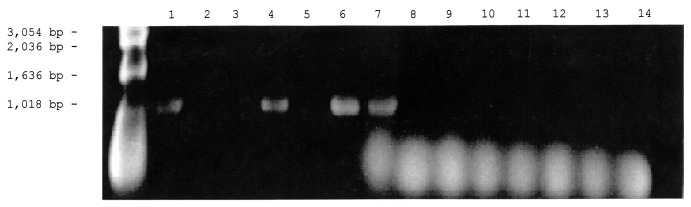
Detection of the *Rickettsia* specific 17-kDa gene by polymerase chain reaction amplification in DNA extracted from ticks and fleas. The vectors were first placed in 1.5-mL microcentrifuge tubes containing 200 µL of 10 mM phosphate-buffered saline, pH 7.4, and were crushed with a micropestle. The suspensions were lysed in 0.5% sodium dodecyl sulfate and incubated with 100 µg/mL proteinase K at 37°C for 1 hour in the case of fleas or overnight in the case of ticks. The lysed suspensions were extracted twice with an equal volume of phenolchloroform, followed by a single chloroform extraction. The extracted DNA was amplified with primer 1 (5′-GCTCTTGCAACTTCTATGTT-3′) and primer 2 (5′-CATTGTTCGTCAGGTTGGCA-3′) as described by Webb et al. (*10*) for amplification of a 434-bp fragment from the rickettsial 17-kDa protein gene. PCR was performed at 30 cycles for 1 minute at 94°C, 5 minutes at 48°C, and 2 minutes at 72°C. The PCR products were then separated by electrophoresis in 1% agarose gel and stained with ethidium bromide. Lanes 1-3: DNA from cat fleas, Lanes 4-6: DNA from dog fleas, Lane 7: 17- kDa gene *Rickettsia felis* DNA (Positive Control), Lanes 8-14: DNA from ticks.

PCR amplifications were done as previously described (*9*) with the DNA extracted from pools of ticks and fleas (Figure 2). Each PCR product was cycle sequenced with the primers described above and fluorescein-labeled dideoxynucleotide bases in the Applied Biosystems model (*11*) DNA sequencing system (ABI, Foster City, CA). Sequences were edited and assembled by using Chromas software (http://www.technelysium.com.au/chromas.html).

To arrive at the most accurate sequence for each PCR product, both forward and reverse sequences were determined. Where differences in nucleotide bases were observed, a predominant base was assigned if most of the sequences contained it. If one base did not predominate, the original chromatographs were consulted to resolve ambiguities. Sequences were compared by using the BLAST software program with 17-kDa sequence from other *Rickettsia* species obtained from the GenBank database. These sequences were aligned for maximal homology by using the Multialign software program (*12*).

## Results

Of the 15 samples of pooled ticks and 6 samples of pooled fleas examined, 3 samples of pooled fleas had the 434-bp product expected for a *Rickettsia* (Figure 2). Nucleotide sequence analysis of the aligned 391-bp segment of 17 kDa confirmed that the three PCR products were identical to each other and to the 17-kDa protein gene of *R. felis* in the database

## Discussion

Recent research on rickettsial diseases in Latin America has included tropical Mexico, Andean Peru, and northern Argentina. The investigation in Minas Gerais State, Brazil, added another ecologic zone and geographic region of Latin America to those in which novel rickettsioses and ehrlichioses have been detected and identified.

BSF is the best-recognized rickettsial disease in Brazil; few reports have been published about human cases of other rickettsioses such as murine typhus and Q fever (*13*). BSF is known to occur in the states of Minas Gerais, São Paulo, Rio de Janeiro, Bahia, and Espírito Santo. Minas Gerais State has a surveillance program for BSF, and since 1990 interest has grown in the study of this disease in areas where residents seeking employment are increasingly exposed to tick-infested habitats. From 1990 to 1994, the incidence of BSF was 0.35 per 100,000 inhabitants, with a higher incidence in the latter half of the year (*13*). The age range most affected was 5 to 14 years (*13*), and the case-fatality ratio was 19% during 1993 to 1995 (*14*).

Our results show that, in addition to *R. rickettsii* and *R. typhi*, *R. felis* is also found in Brazil, as indicated by positive serology in human cases (*8*). Our data are the first indication by PCR of the presence of *R. felis* in fleas from Brazil. The *Ctenocephalides* spp. flea is proposed as a possible vector of this new rickettsial disease in Brazil.

Because of the complicated differential diagnosis of febrile exanthems, which includes dengue fever and other viral, rickettsial, and bacterial diseases, more attention should be paid to diagnostic laboratory investigation of rickettsial diseases. *R. felis* has been identified as the etiologic agent of a new rickettsiosis whereever it has been investigated: United States (Texas), Mexico, Brazil, and France (*8*). In Latin America, two reports have been published of human rickettsioses caused by *R. felis* in Mexico and Brazil (*7*,*8*). In both these reports, neurologic involvement was described, suggesting a severe clinical course associated with *R. felis* (*15*).

Although our investigation does not provide evidence for widespread flea infection by *R. felis* in Brazil, we demonstrate for the first time the presence of infection by this bacteria in Brazilian *Ctenocephalides* fleas. The descriptions of *R. felis*-positive human cases (*8*) in the same area where *R. felis* was identified in *Ctenocephalides* fleas indicate the possibility of this flea’s being the vector of human *R. felis* rickettsiosis in Brazil.
